# Hyperglycemia disturbs trophoblast functions and subsequently leads to failure of uterine spiral artery remodeling

**DOI:** 10.3389/fendo.2023.1060253

**Published:** 2023-04-05

**Authors:** Yueyue Zhu, Xiaorui Liu, Yichi Xu, Yi Lin

**Affiliations:** ^1^ Reproductive Medicine Center, Shanghai Sixth People’s Hospital Affiliated to Shanghai Jiao Tong University School of Medicine, Shanghai, China; ^2^ The International Peace Maternity and Child Health Hospital, School of Medicine, Shanghai JiaoTong University, Shanghai, China

**Keywords:** hyperglycemia, trophoblast, decidual NK cells, Hofbauer cells, uterine spiral artery remodeling

## Abstract

Uterine spiral artery remodeling is necessary for fetal growth and development as well as pregnancy outcomes. During remodeling, trophoblasts invade the arteries, replace the endothelium and disrupt the vascular smooth muscle, and are strictly regulated by the local microenvironment. Elevated glucose levels at the fetal-maternal interface are associated with disorganized placental villi and poor placental blood flow. Hyperglycemia disturbs trophoblast proliferation and invasion *via* inhibiting the epithelial-mesenchymal transition, altering the protein expression of related proteases (MMP9, MMP2, and uPA) and angiogenic factors (VEGF, PIGF). Besides, hyperglycemia influences the cellular crosstalk between immune cells, trophoblast, and vascular cells, leading to the failure of spiral artery remodeling. This review provides insight into molecular mechanisms and signaling pathways of hyperglycemia that influence trophoblast functions and uterine spiral artery remodeling.

## 1 Introduction

Hyperglycemia (HG) is a common metabolic imbalance in pregnant women with Type 1 diabetes mellitus (T1DM), Type 2 diabetes mellitus (T2DM), or combined with pregnancy and gestational diabetes mellitus (GDM) (1). Abnormally high blood glucose levels in the pregnancy may lead to abnormal uterine glucose concentration (2). Unhealthy dietary habits, such as a high glucose intake, are prevalent nowadays. Both HG and HG-induced cytokines releasing affect trophoblast function and uterine spiral arteries (SAs) remodeling, which can in turn increase the incidence of pregnancy complications, such as pre-eclampsia, malformations and miscarriage, and thus endanger the health of pregnant women and fetuses (3, 4).

SAs facilitate the exchange of nutrients, gases, and waste between mother and fetus (5). During SA remodeling, the original uterine SA converts into low-resistance and highly dilated vessels to meet the pregnancy blood requirements and prevent damage to the villi (6). Uterine SA remodeling has four stages. Firstly, trophoblasts and leukocytes in the vessel wall are not yet invasion, the endothelial cells (ECs) and smooth muscle cell layers of the vessel wall are intact. Secondly, the vascular structure begins to be destroyed by decidual NK (dNK) cells and macrophages in the vessel wall before trophoblast invasion (7). Thirdly, extravillous trophoblasts (EVTs) appear in the vessel wall and lumen. Finally, vascular smooth muscle cells (VSMCs) and ECs are completely lost and replaced by intravascular EVTs, and the wall matrix is replaced by fibrin-like substances. In addition, cytokines, angiogenic factors, enzymes, and extracellular matrix (ECM) also participate in regulating SA remodeling (8, 9). Trophoblasts are exposed to the maternal circulation and are influenced by the maternal endocrine, metabolic, and inflammatory environments. Hence, the influence of various maternal microenvironmental factors, such as high fat, high sugar diets, obesity and diabetes may affect trophoblast functions and the SA remodeling (10).

There are fewer blood vessels and villi in placenta of diabetic women with unexplained stillbirths than those with live births (11). Different from being replaced by trophoblasts in normal pregnancy, VSMCs in the placenta of biobreeding diabetes-prone rat were almost complete while SA remodeling was failure (12). Taken together, HG may cause insufficient SA remodeling *via* impaired trophoblasts. Herein, we will summarize the current knowledge on the possible effects of HG on trophoblast function as well as their role in uterine SA remodeling.

## 2 Trophoblasts and uterine SA remodeling

Trophoblasts are the first cell type to differentiate during embryogenesis. In this process, trophoblast stem cells are derived from embryonic trophectoderm. They can differentiate into various trophoblast cell lines and acquire many specialized functions, including invasion potential and endocrine activity (13). Cytotrophoblasts (CTBs) are stem cells that proliferate rapidly once embedded in maternal decidua. The outer layer of CTBs fuses into primitive syncytiotrophoblasts (STBs), which erode surrounding decidua and generate lacunae filled with blood (14). Placental villi bathed in maternal blood are floating villi in charge of placenta material transport, whereas villi, anchored in the placental basal plate, differentiate into EVTs (15).

**Figure 1:**
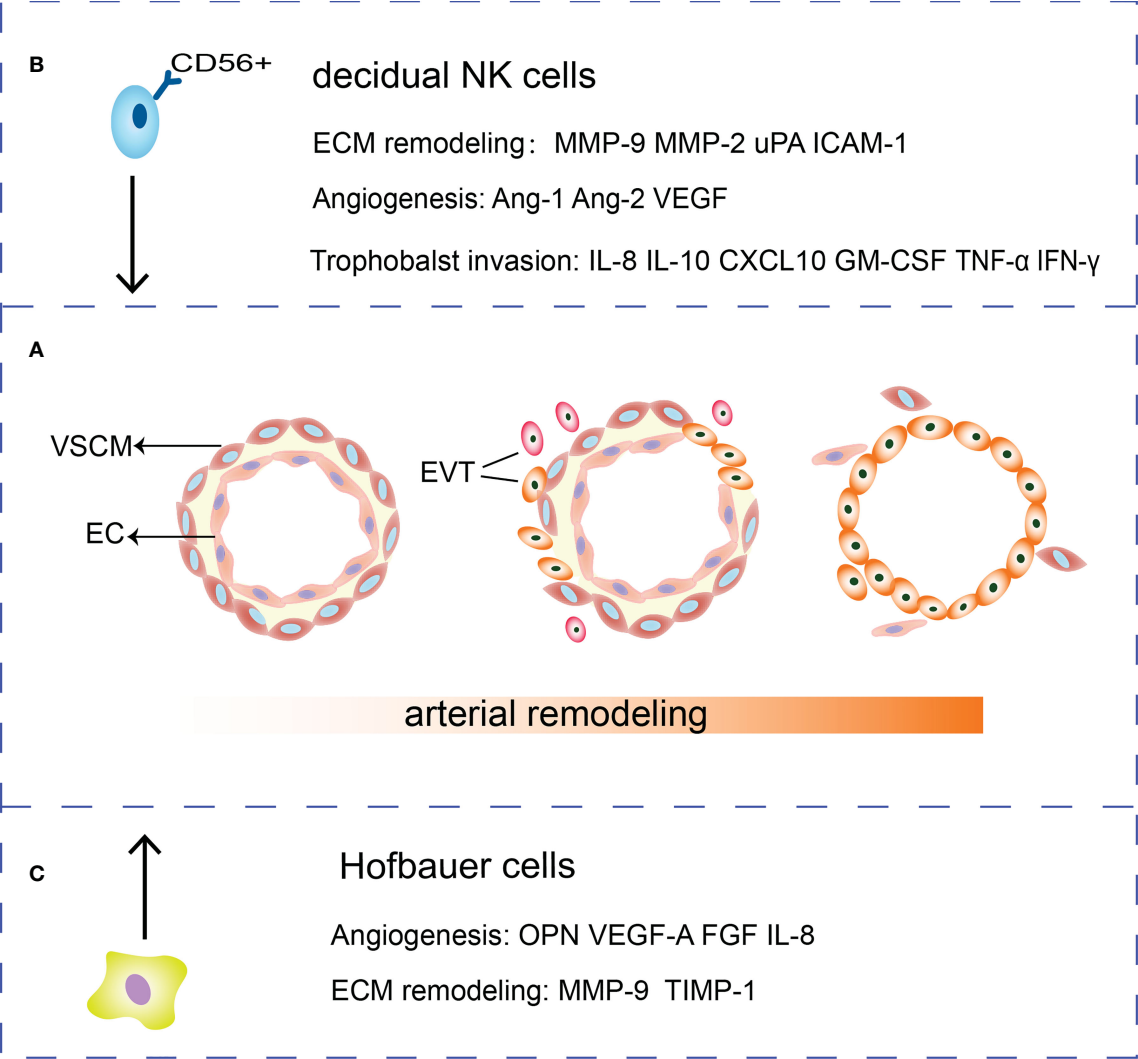
Roles of HBCs, EVTs and dNK cells in SA remodeling. **(A)** During the SA remodeling, VSMCs surrounding the arteries are removed and ECs are gradually replaced by EVTs. **(B)** dNK cells secrete cytokines such as IL-10, GM-CSF, TNF-α, and IFN-γ and chemokine IL-8, CXCL10 to regulate trophoblasts invasion. Meanwhile, dNK cells are potent sources of angiogenic factors such as Ang-1, Ang-2, VEGF. dNK cells secrete MMP-2, MMP-9, uPA and ICAM-1 that participate in ECM remodeling. **(C)** HBCs can secrete MMP and TIMP to remodel the extracellular matrix. Besides, HBCs secrete a range of factors that play a role in remodeling vessels such as OPN, FGF, VEGF-A and IL-8.

During differentiation, trophoblasts lose adherent epithelial phenotype and acquire a mesenchymal phenotype and invasion ability through epithelial-mesenchymal transition (EMT). The E-cadherin/β-catenin complex is a calcium-dependent transmembrane protein distributing in epithelial tissues which form cell tight junctions, inhibit cell movement and maintain epithelial integrity (16). Decreased E-cadherin expression results in the upregulation of integrin α1β1, α5β1 and α_V_β_3_, VE-cadherin, intercellular adhesion molecule-1 (ICAM-1), and vascular cell adhesion molecule-1(VCAM-1) (17, 18). EVTs invade the decidual stroma to form the interstitial extravillous trophoblasts (iEVTs) to promote the muscular layer of vessel wall degradation (19). EVTs invade the decidual blood vessels to form the endovascular extravillous trophoblasts (enEVTs) to replace ECs and VSMCs (20). VSMCs undergo morphological changes while EVTs penetrate the vessel wall *via* intravascular or interstitial pathways. They shift to a synthetic phenotype, migrate from the vessel wall, and undergo apoptosis. Trophoblasts secrete platelet derived growth factor BB (PDGF-BB) to bind the PDGF receptor β (PDGFR-β) of VSMCs to activate the PDGF signaling pathway and induce de-differentiation of VSMCs (21). EVT secretes tumor necrosis factor α (TNF-α), tumor necrosis factor-related apoptosis-inducing ligand (TRAIL), and Fas ligand to induce apoptosis of VSMCs (22) (**Figure 1A**).

In addition, trophoblast-immune cell-vascular interactions are important determinants of adequate SA remodeling. The dNK cells, macrophages, trophoblasts, and their crosstalk are important for adequate SA remodeling, and any dysregulation may lead to remodeling obstacles (23, 24).

## 3 HG affects the biological functions of trophoblasts in uterine SA remodeling

Proliferation, signaling disorders, impaired placenta blood flow, and increased vascular resistance were observed in streptozotocin-induced GDM rat model (25–27). And decreased VSMC apoptosis was observed in placentas of mice with GDM (28). In addition, HG impaired the differentiation of trophoblast stem cell into an invasion phenotype and inhibited the trophoblast invasion, which further demonstrated that HG directly alters trophoblast lineage development (10). In the following paragraphs, we discuss in depth how HG influenced trophoblasts proliferation and invasion.

### 3.1 HG damages trophoblasts proliferation involved in uterine SA remodeling

Highly proliferating trophoblasts are necessary for placenta formation. And cell cycle control is very important in the proliferation process. *In vitro* studies have demonstrated that HG induced cell cycle arrest at G0/G1 in human trophoblast BeWo, JAR and HTR-8 cells, indicating that HG has the potential to inhibit trophoblasts proliferation (29, 30). Transcriptome and metabolome analysis showed that HG perturbed the phosphatidylinositol phosphate signaling pathways that involved in cell proliferation in BeWo cells (31). HG may inhibit cell proliferation by regulating the process of translation *via* epigenetic modifications, such as non-coding small molecule RNAs (32). HG upregulated the expression of miR-137, resulting in a negative modulatory effect on AMP-activated protein kinase, which ultimately stimulated the expression of IL-6 to inhibit cell proliferation (33). HG also promoted the expression of miR-136, inhibited the trophoblasts proliferation by suppressing E2F1 which is an important cell cycle regulator mediating the G1/S transition (34). MiR-362-5p was downregulated under HG conditions and inhibited the PI3K/AKT pathway by upregulating glutathione-disulfide reductase (GSR) directly, ultimately leading the inhibition of HTR-8 cells proliferation (35). MiR-520h was upregulated and inhibited cell proliferation by downregulating mTOR expression in HG-treated HTR-8 cells (36).

*In vivo*, immature villi in human diabetic placentas in term pregnancies suggested that HG provided more nutrition for continuous cell growth but delayed the cell differentiation and maturation (37). Upregulated Ki67 was observed in the term placenta of patients with GDM (38). However, Ki67 was downregulated in first-trimester human placental tissue with T1DM (39). In rat, the proliferative capacity of trophoblasts was weakened and the number of Ki67 positive cells decreased as the gestational day increases. At day 17 of pregnancy, Ki67 positive cells was higher in diabetic rat placentas than normal control (40). The different effects of HG on the proliferation of trophoblasts depend on gestational periods. HG provided excess nutrients for cell growth at the end of pregnancy, which also could explain why women with diabetes had higher placenta weight. Conversely, HG inhibited trophoblast proliferation in the first trimester because this period of placental development is particularly susceptible to environmental perturbations and any changes in the microenvironment may lead to impairment of trophoblast function (41).

### 3.2 HG damages trophoblasts invasion involved in uterine SA remodeling

In the progress of trophoblasts invasion into decidual tissue, EVTs produce proteases, such as fibrinogen activation system enzymes, matrix metalloproteinases (MMPs), and tissue inhibitor of metalloproteinases (TIMPs), to regulate the ECM remodeling and trophoblast invasion (42). The fibrinogen activation system comprises fibrinogen activators, such as urokinase-type plasminogen activator (uPA), and enzyme inhibitors such as fibrinogen activator inhibitor type 1 (PAI-1) (43). MMPs are a family of more than 23 zinc-binding enzymes, exhibiting proteolytic activity promoting trophoblast invasion to the uterine wall. MMP activity is mainly regulated by TIMP. MMP2 and MMP9 are the most important MMP enzymes involved in trophoblasts during the first trimester (44). Pro-uPA zymogen activates uPA after binding uPAR. Activated uPA in turn cleaves and activates MMPs as well as degrades local matrix protein (45). Exposure to excess glucose may lead to shallow trophoblast migration and invasion, leading to abnormal uterine SA remodeling.

*In vitro*, trophoblast cell invasion is adversely disturbed under HG condition (46–51). Belkacemi et al. showed that trophoblasts invasion was reduced by approximately 62% and the activity of uPA was lower when HTR-8 cells were treated with 10mM of glucose (52). Furthermore, uPA in human early pregnancy trophoblast cell Sw.71 also decreased with the increasing glucose concentration (53). The increased E-cadherin, decreased Twist1 and Vimentin in HTR-8 cells under HG indicated a failure of EMT. EMT not only participates in EVT invasion but also balances the CTB-EVT differentiation (54). It was reported that MiR-137 was elevated in HG-treated HTR-8 cells, and the upregulation of miR-137 decreased the expression of fibronectin type III domain-containing 5, thereby inhibited the viability and migration of HTR-8 cells (55).

Some *in vitro* studies has shown that HG promoted the invasion of trophoblasts. HG induced proteoglycans alterations in 3A-Sub-E cells which is isolated from human full-term placenta, followed the increased MMP-2 and MMP-9 and decreased TIMP-2 (56). However, the HG altered proteoglycans on the surface of trophoblasts can lead to ECM deposition and complications in diabetic placenta (57). Normally, physiological levels of reactive oxygen species (ROS) promote angiogenesis, and the placental antioxidant system prevents ROS overproduction (58). HG induced the expression of the Cytochrome P450 enzyme family 1, subfamily B, polypeptide 1 (CYP1B1) which promoted trophoblast migration *via* MMP2. Inhibition of CYP1B1 may suppress ROS production under HG condition, which may provide a new method for diabetic complications caused by ROS overload (59). In placentas of diabetic rats at mid-gestation, increased ROS triggers trophoblast spreading with the increased expression of MMP-2 and MMP-9 (60).

Collectively, it is not difficult to suppose that HG inhibits the invasion and migration of trophoblasts derived from the first trimester, but promotes the invasion and migration of trophoblasts derived from the third trimester. Primary trophoblasts isolated from human placentas culture under HG could further verify our inference.

### 3.3 HG alters oxygen tension in placenta during uterine SA remodeling

Before the first 10 weeks of gestation, EVT forms a trophoblast plug to prevent maternal blood from entering the intervillous space and creates a physiologically hypoxic environment (2%–3% O_2_) (61). The hypoxia-inducible factor 1 (HIF-1) plays a transcriptional regulatory role in hypoxic environment. There is an increased expression of TGF-β under hypoxia, thereby inhibiting trophoblasts differentiation (62). At the 12^th^ week of gestation, the trophoblast plug dissolves and uterine SA begins to remodel, following a gradually increased oxygen concentration (8% O_2_) around the trophoblast (63). Both HIF-1α and TGF-β expression decreases with increasing oxygen concentration, enabling trophoblast differentiation and ensuring extensive EVTs invasion into SA with increased MMP9 (64). However, after trophoblast differentiation into mature EVT, hypoxia and elevated HIF can promote EVT invasion (65, 66).

HG increased the thickness of trophoblast membranes and the massive collagen deposition, resulting in altered oxygen gradients in placenta and local hypoxia at the maternal-fetal interface (40). Downregulation of miR-29b in placenta with GDM promoted trophoblast invasion by upregulating the expression of HIF3A (67). Hypoxia promotes the invasion of mature EVTs. It is reasonable to suppose that HG promotes the invasion of trophoblasts in the third trimester placenta. It is also suggested that mild HG increased capillaries through negative feedback regulation of ischemia and hypoxia, however, sustained severe HG triggered hypoxia/ischemia and inhibited vascular endothelial growth factor (VEGF)/VEGFR-2 binding, thereby reducing excessive capillary formation (68, 69). What is more, HG can alter trophoblasts development by blunting trophoblast stem cell responses to low oxygen levels (10).

### 3.4 HG disrupts trophoblasts releasing angiogenic factors

Trophoblasts secrete angiogenic factors during uterine SA remodeling. VEGF disrupts the VSMC and ECs. Placental growth factor (PlGF), prominently expressed in villous CTBs and STBs, promotes angiogenesis under hypoxic conditions (70). Angiopoietins (Ang1, Ang2) and their receptor Tie-2 play an important role in stabilization or breakdown of blood vessel (71). Fibroblast growth factor (FGF) and PDGF-BB are involved in vasculogenesis and angiogenesis (72). Anti-angiogenic factors, such as soluble fms-like tyrosine kinase-1 (sFlt-1) and soluble endoglin (sEng), are secreted. SFlt-1 is the soluble form of VEGFR-1, with a high affinity for VEGF, but no signal transduction function (73). Besides, sEng interferes with transforming growth factor β (TGF-β) and inhibits endothelial nitric oxide synthase activation, thereby disrupting angiogenesis (74).

In the first trimester trophoblasts HTR-8 and SW.71, HG decreased the secretions of VEGF, PlGF and uPA, while increased the secretions of anti-angiogenic factors sFlt-1 and sEng to inhibit artery remodeling (48, 52, 53, 75, 76). In placenta of women with GDM, increased VEGF, Ang, Eng and endothelin may lead to a collapse between angiogenic and anti-angiogenic factors (77). However, mild HG did not change the expression of VEGF (78). Persistent HG might thicken the placenta, increased the expression of HIF, thereby promoting the expression of angiogenic factors, such as VEGF and PIGF (79). FK506-binding protein like, acting as an anti-angiogenic protein and a regulator of inflammation, decreased in T1MD placenta and trophoblast cell line ACH-3P treated with HG under hypoxia condition (80). HG also promoted MT1-MMP and angiogenesis *via* PI3k signaling in GDM placenta (81). Despite these conflicting reports, it is certain that the balance between angiogenic and anti-angiogenic factors is disrupted under HG. HG in the first trimester inhibits uterine SA remodeling by inhibiting the proliferation, invasion, and migration of trophoblasts. HG may cause hypercapillarization of villi due to collagen deposition caused by hypoxia and abnormal trophoblasts migrations in the third trimester, but these vessels are immature (82).

## 4 HG affects the crosstalk between immune cells and trophoblasts

Pregnant uteri are colonized by large number of immune cells, the most abundant cells of which are dNK cells and macrophages, followed by T cells and dendritic cells. Approximately 75% of decidual leukocytes are CD56^bright^CD16^-^ dNK cells and are not cytotoxic (83). Decidual macrophages, recruited from the maternal circulation, are polarized toward M1 macrophages during peri-implantation period while a profile of a mixed M1 and M2 type during EVTs invading the SA (84). Different from decidual macrophages, Hofbauer cells (HBCs) are the villous macrophages in the stroma of the first-trimester placenta arising from hematopoietic stem cells and are characterized as CD14^+^ CD68^+^ cells (85).

Chemokines and their receptors also play important roles in trophoblast migration and immune cells recruitment at the maternal-fetal interface (86). The dNK cells secrete IL8, CXCL10, TNF, interferon (INF) γ, TGF-b, and angiogenic factors such as VEGF-A, VEGF-C and PlGF (15, 87). Trophoblasts express the IL8 receptor CXCR1, the CXCL10 receptor CXCR3, TNF receptor TNFR1, as well as VEGFR-1 and VEGFR-3. Trophoblasts produce human leukocyte antigen to increase the levels of inhibitory receptors in dNK cells, maintaining their inactive phenotype (CD16^−^CD56^+^) (88). Meanwhile, macrophages secret IL-33, granulocyte colony-stimulating factor (G-CSF), CXCL1, TGF-b, TNF-α and Wnt5a to regulate trophoblasts invasion and migration (89). Immune cells, interacting with ECs, fibroblasts, and trophoblasts, promote the SA remodeling and placental growth. Any dysregulation of these factors may lead to remodeling obstacles (15).

*In vitro*, HG could mediate trophoblast releasing inflammatory factors IL-1β, IL-4, IL-8, and IL-6, IFN-γ, TNF-β, CXCL1 and G-CSF, indicating that HG created a pro-inflammatory environment at the maternal-fetal interface (76, 90, 91). High level of pro-inflammatory TNF-α was found both in GDM and TD2M placenta. Decreased IL-4 was found in T2DM and MGH placenta, promoting NK cell into active phenotype (92). This also reminds us that maternal HG caused by diabetes mellitus can lead to a disturbance in the balance of pro-inflammatory and anti-inflammatory factors at the maternal-fetal interface. In the following sections, we discuss in depth how HG disturbed immune cells and lead to the failure of SA remodeling.

### 4.1 HG affects crosstalk between dNK cells and trophoblasts

The dNK cells and EVTs interact with vascular ECs to promote SA remodeling (93). Firstly, dNK cells induce the apoptosis of VSMC and ECs, destruct blood vessel and secrete Ang-1, Ang-2, VEGF and MMPs to mediate angiogenesis (94, 95). The dNK cells secrete MMP-2, MMP-9, uPA, adhesion molecules such as ICAM-1 to regulate ECM remodeling (96–98). DNK cells express killer immunoglobulin receptor (KIR), CD94/NKG2A, and immunoglobulin like transcripts 2 (ILT2). These three receptors can interact with HLA-C, HLA-E and HLA-G on trophoblasts respectively, regulating trophoblast invasion (87). Cytokines, such as IL-10 and granulocyte-macrophage colony-stimulating factor (GM-CSF) and chemokines IL-8, CXCL10 produced by dNK cells could promote EVTs invasion while the cytokines TNFα and IFN-γ inhibited trophoblast invasion by upregulating PAI expression (87, 99). IL-8 can also increase trophoblast expressing integrins α1 and β5 to gain an invasive phenotype (100) (**Figure 1B**).

A test for GDM peripheral blood showed a higher percent of cytotoxic NK cells (CD16^+^CD56^dim)^ in the GDM group than in controls (101). Some scholars believe that dNK cells are derived from the recruitment of peripheral CD56^bright^ NK cells, which acquire dNK cells phenotype under the influence of a specific decidual microenvironment (102). Thus, changes in peripheral blood NK cells may lead to changes in the decidual NK cells. Fewer CD56^+^cells adhere to decidual endothelium, while more diabetic CD56^+^ cells adhere to pancreatic endothelium in pregnant women with T1DM and T2DM, indicating that HG impairs egression of CD56^+^ cells into the decidua (103). Cytotoxic CD16^+^ CD56^−^NK cell both increased in maternal blood and placenta extravilli of GDM and T2MD. Placental CD16^-^CD56^+^ NK cells were higher in GDM and lower in T2DM, irrespective of region (92). GDM and T2MD are characterized by excessive insulin resistance, followed by maternal HG, triggering a “glucose stress” response and concurrent systemic inflammation (104). This response involves altered infiltration, differentiation, and activation of maternal innate and adaptive immune cells, which may explain increased CD16^+^ NK cells. The control of blood sugar affects the expression of cytokines. Besides, cytokines differ in recent-onset DM and long-standing DM (105, 106). Thus, we don not exclude glycemic control conditions and duration of maternal HG are responsible for CD56^+^ NK cells percentage and cytokine levels different in GDM and T2DM. In addition to the phenotypic changes of NK cells, the cytokines secreted by NK cells also change. Simultaneously, CD56^+^ cells producing TGF-β and VEGF decreased significantly in patients with GDM (107). This secretory change may affect the regulation of trophoblast migration and invasion by dNK cells in high-risk pregnancy (108). Above all, HG may decrease dNK in the uterine wall, leading to a diminished interstitial trophoblast invasion and less SA remodeling.

### 4.2 HG affects crosstalk between macrophages and trophoblasts

Adopting an M2 polarity phenotype, HBCs express TIMP-1, MMP9, VEGF-A, osteopontin (OPN), and FGF to affect ECM and vascular remodeling (109–111). HBCs also secrete inflammatory factors such as IL-8, CCL-2, CCL-3, and CCL-4 with proangiogenic properties (109) (**Figure 1C**). Moreover, CD14^+^ macrophages in early pregnancy decidua induce the breakdown of ECM and phagocytose apoptotic VSMCs to remodel the uterine SA (112).

HBCs treated by HG switched their M2 polarity profile towards M1 phenotype, which is not conducive to angiogenesis (113). M2 macrophages involve in anti-inflammatory processes and promote angiogenesis and tumor progression, which can produce protease to degrade the ECM (114). Thus, a reduction in the number of M2 phenotype cells may lead to impaired vascular remodeling. However, Schliefsteiner et al. showed HBCs maintain their M2 polarization to maintain a successful pregnancy, even in inflammatory states such as GDM. The co-cultivation of HBCs from GDM placentas and placental arterial endothelial cells (pAECs) did not alter ECs activation (115). Zhang et al. reported that M2a macrophages, majoring in tissue repair, increased in villi and more collagen was deposited in uncontrolled T2DM group compared with the healthy group (116). All in all, HG can disturb the balance between pro-inflammatory and anti-inflammatory subtypes of HBCs, which may cause adverse pregnancy outcomes.

## 5 Limitation and future direction

A recent study isolated SA from 12 to 23 weeks of gestation and found that the vascular remodeling was not complete until 23 weeks of gestation (117). Previous studies have mainly focused on pregnant women with GDM with few studies focusing on pregnant women with T1DM or T2DM. GDM is mainly screened at 24-28 weeks of gestation, but hyperglycemia occurs before 24 weeks. In addition, early GDM may has worse pregnancy outcomes (118). Therefore, the molecular and signaling pathway changes in the placenta of patients with GDM are also valuable for understanding the effect of HG on the uterine SA.

However, previous *in vivo* studies also had some limitations. First, the number of placenta cases in these studies is relatively small and the individual differences in patients are large. Secondly, no information was discussed on medication of women in the case group. Another limitation is lack of protein involved in ECM remodeling and angiogenesis such as MMP2, MMP9, uPA, PIGF and VEGF. SA remodeling occurs mainly before 24 weeks, therefore, staining of above protein in the first and second trimester placenta villi is more indicative of the effect of HG on trophoblast function.

All previous *in vitro* studies differed in terms of glucose dose, treatment time and cell line. Different or opposite conclusions have been drawn regarding the effect of HG on trophoblast function and uterine SA remodeling. For example, Basak et al. reported tube formation substantially increased at 25-30mM glucose and decreased at 40mM glucose in HTR-8 cells (119). In addition, McLeese et al. pointed out that HTR-8 cells did not survive in 5 mmol/L glucose over 48h, possibly due to the rapid glucose consumption (120). Inadera et al. pointed out that when BeWo cells were cultured at physiological levels of 5 mM glucose, the cells detached from dishes (31). Thus, the above researches remind us choosing appropriate glucose concentration is necessary to study the effects of HG on trophoblasts biological behavior and SA remodeling. The *in vitro* experiments used in this review are summarized in **Table 1**.

**Table 1:** The *in vitro* experiments used in this study are summarized.

Reference	Phenotype	cell line	key molecule	pathway	glucose concentration
proliferation					
29	HG inhibited the proliferation of first-trimester trophoblast	BeWo, JAR, JEG-3	Cyclin B1↓	/	5mM VS 25mM
30	HG inhibited the proliferation and arrested trophoblast in G0/G1 phase	HTR-8	PCNA↓	circ_FOXP1/miR-508-3p/SMAD2	5.5mM VS 30mM
31	HG perturbed biochemical networks *via* elevated oxidative stress	BeWo	/	/	11mM VS 25mM
32	HG inhibited cell proliferation	HTR-8, BeWo	/	miR-132/PENT	5mM VS 25mM
33	HG inhibited HTR-8 viability and proliferation	HTR-8	/	miR-137/PRKAA1/IL-6	5mM VS 25mM
34	HG inhibited cell proliferation	HRT-8, BeWo	E2F1↓	miR-136/E2F1	5mM VS 25mM
35	HG inhibited HTR-8 proliferation and induced apoptosis	HTR-8	/	MiR-362-5p/GSR/PI3K/AKT	5mM VS 25mM
36	HG inhibited HTR-8 proliferation and induced apoptosis	HTR-8	/	miR-520/mTOR	5.5mM VS 25mM
39	HG reduced trophoblast proliferation	Primary trophoblasts	ki67↓	/	5.5mM VS 25mM
Invasion, migration, and angiogenesis					
46	HG inhibited cell proliferation and migration	HTR-8	/	miR-134-5p/FOXP2	5mM VS 25mM
47	HG suppresses trophoblast viability,migration and induces apoptosis	HTR-8	/	circ-PNPT1/miR-889-3p/PAK1	5mM VS 25mM
48	HG inhibited HTR-8 viability, migration, and invasion	HTR-8	/	PLGF/ROS	0, 10, 15, 20, 25, 30 μM
49	HG inhibited cell migration and promoted apoptosis	HTR-8	/	FOXC1/FGF19/AMPK	5mM VS 25mM
50	HG inhibited cell viability, migration, and invasion, and promoted cell apoptosis	HTR-8	/	CTRP6/PPARγ	5.5mM(control), 10mM, 20mM, 30mM
51	HG enhanced HTR-8 autophagy and reduced invasion	HTR-8	LC3-II↑, p62↓	/	5mM VS 30mM
52	HG inhibited HTR-8 invasion	HTR-8	uPA↓	/	2.5mM VS 5mM and 10mM
53	HG inhibited Sw.71 invasive profile	Sw.71	uPA↓; VEGF, PIGF↓; sENG, sFIt-1↑	/	45(control), 135, 225, 49, 945mg/dl
54	HG inhibited HTR-8 EMT	HTR-8	E-cadherin↑, Vimentin, Twist1↓	ST2/PI3K/AKT/AMPK; ST2/P62/Twist	5mM VS 30mM
55	HG inhibited HTR-8 invasion	HTR-8	/	miR-137/FNDC5	5mM VS 25mM
56	HG stimulated trophoblast invasion and angiogenesis	3A-Sub-E	MMP-2, MMP-9↑, TIMP-2 ↓	/	5.6mM VS 30mM
59	HG stimulated trophoblast invasion and migration	HTR-8	/	/	normal medium VS 20mM
75	HG induced anti-angiogenic signaling in CTBs	Sw.71	VEGF, PIGF↓; sENG, sFIt-1, IL-6↑	/	100 (control), 150, 200, 300, or 400 mg/d
76	HG induced anti-angiogenic, and anti-migratory in first trimester trophoblast cells.	Sw.71	sENG, sFIt-1↑	/	5mM(control), 10mM, 25mM, 50mM
80	HG cause aberrant angiogenesis profile	ACH-3P	FKBPL↓, SIRT-1↓, PlGF↑	/	5mM VS 25 mM
119	HG promoted tube formation at 25 mM and inhibited tube formation at 40mM	HTR-8	MMP9 ↑	/	5.5 (control), 11, 25, and 40 mM

Although the relevant molecular mechanisms and signaling pathways of HG influencing trophoblast functions have been reported in these studies, the studies have mostly focused on cell lines and animal models. However, cell lines do not truly reflect *in vivo* conditions. Information obtained from animal models is also limited because SA remodeling differs between human and rats (121, 122). Therefore, it is crucial to establish a suitable model with appropriate sugar concentration for further study. *Ex vivo* model, such as human placenta-decidua co-culture, can also be used to quantify the extent of SA remodeling (123). In addition, human trophoblast organoids show similar cellular composition and biological behavior to those of immature human placentas. Despite the lack of such studies, we believe that human trophoblast organoid models cultured under HG conditions will help us understand the effect of HG on the remodeling of uterine SAs (124).

## 6 Conclusion

**Figure 2:**
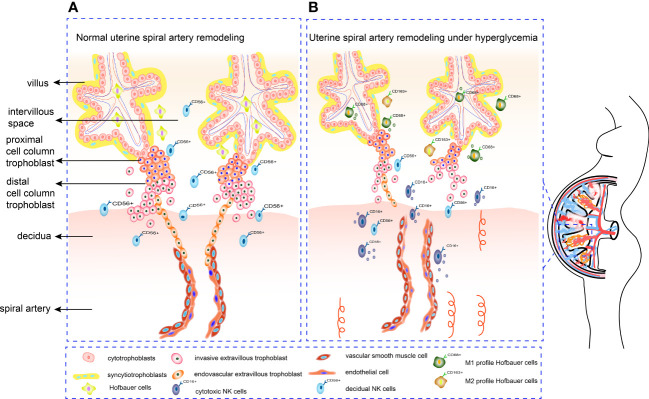
Mechanism of SA remodeling in normal pregnancy and hyperglycemia pregnancy. **(A)** CTBs proliferate rapidly once embedded in maternal decidua. The outer layer of CTBs fuses into primitive STBs, which can form proliferative proximal cell column trophoblasts. EVTs differentiate from distal cell column and break through the overlying STB layer, detaching from distal cell columns, migrating into the decidual stroma, and remodeling the SA. **(B)** Under hyperglycemia conditions, the proliferation and invasion ability of trophoblasts alters. Increased cytotoxic CD16^+^CD56^dim^ NK cells can form an inflammatory environment. Meanwhile, HBCs switch their M2 polarity profile towards M1 phenotype, which is not conducive to angiogenesis. Deficient artery transformation and immature new blood vessels can be observed in hyperglycemic placenta.

Uterine SA remodeling requires appropriate trophoblast proliferation, invasion, and tissue remodeling, which involves a balanced MMP, TIMP and uPA. Meanwhile, trophoblasts, dNK cells and HBCs can secrete serious cytokines and angiogenic factors to regulate SA remodeling. Crosstalk between immunity cells and both trophoblast and vascular cells at maternal-fetal interface is also a part of the remodeling process. Inappropriate glucose concentrations may lead to abnormal trophoblast proliferation, migration, and invasion by disrupting the balance between MMP and TIMP. In addition, HG disrupts the balance between angiogenic factors Ang-1, VEGF, PlGF and anti-angiogenic factors sFlt-1 and sEng. Furthermore, an impaired immune cell profile under HG conditions influences SA remodeling (**Figure 2**). Understanding how HG affects SA remodeling by influencing trophoblast function is crucial for revealing the mechanisms by which diabetes leads to pregnancy complications and adverse pregnancy outcomes. This review may provide a theoretical basis for future foundation and clinical research.

## Author contributions

YZ: Search literature and write original draft. XL: Review, editing, project administration. YZ and XL contributed equally to this work. YX: Review and editing. YL: Funding acquisition, Resources, Supervision, Writing – review and editing. All authors contributed to the article and approved the submitted version.
